# The Role of *SLPI* Gene-Mediated Inflammation in Osteoarthritis

**DOI:** 10.3390/biom15070909

**Published:** 2025-06-20

**Authors:** Mahmuda Siddika Shefa, Wanil Kim

**Affiliations:** Department of Biochemistry, Institute of Medical Science, School of Medicine, Gyeongsang National University, Jinju 52727, Republic of Korea; shefarahman016@gmail.com

**Keywords:** osteoarthritis, SLPI, inflammation, collagen, cartilage, tissue degeneration, extracellular matrix

## Abstract

Osteoarthritis (OA) is a degenerative disease of joint tissue characterized by the breaking down of cartilage and resulting changes in synovium and bone. Mechanics and biology interact in a feed-forward manner in that imbalanced joint loading leads to tissue degeneration and vice versa. Amid numerous genetic factors, the Secretory Leukocyte Protease Inhibitor (*SLPI*) gene encodes a protein that plays a crucial role in inhibiting proteases, modulating inflammation, promoting tissue repair, and regulating immune responses. In the context of OA, SLPI has been identified as a key regulator in joint homeostasis. The release of SLPI in human tissues is augmented by pro-inflammatory factors. Such factors include cytokines released during infection or inflammatory processes, such as interleukins-1 and 6 (IL-1 and IL-6), and tumor necrosis factor alpha (TNF-α) released in many inflammatory rheumatic diseases. In this work, a comprehensive review of SLPI-mediated inflammation in OA, its biological functions, and its association with OA is described, providing a foundation for future investigations into its potential therapeutic use. As there is no effective strategy to treat or prevent OA in clinic, further investigation is encouraged to explore the translational possibility of SLPI for drug development.

## 1. Introduction

Articular cartilage is an avascular tissue where nutrient diffusion—facilitated by the exchange of polymers, oxygen, glucose, and lactate—is essential for maintaining chondrocyte viability. However, with age, the progressive loss of glycosaminoglycans impairs this diffusion, especially in the deeper cartilage zones, leading to poor nourishment and an increased risk of osteoarthritis (OA) [1]. Normally, cartilage ensures smooth joint movement and shock absorption, but its degradation, exacerbated by aging, diabetes, obesity, and injury, accelerates joint degeneration [2].

Current OA therapies mainly alleviate symptoms without altering disease progression [3]. While some investigational drugs show promise in modulating inflammation and pathology, most remain in early clinical trials and are not yet widely available [3,4,5]. OA’s pathogenesis comprises a complex interplay of biochemical, biophysical, and epigenetic mechanisms, many of which are still not fully understood [2,6]. Recent research into the osmotic, mechanical, and electrical environment of chondrocytes has enhanced our understanding of cartilage homeostasis and the potential for tissue-engineered models in drug development [7,8]. Yet, no disease-modifying OA treatments have been approved to date.

One emerging target of interest is the Secretory Leukocyte Protease Inhibitor (SLPI), an ~11.7 kDa protein with two homologous domains and 86% identity between bovine and human forms. A member of the whey acidic protein family, SLPI was initially identified as a protease inhibitor of neutrophil elastase and cathepsin G but is now recognized for its broader roles, including antimicrobial, wound-healing, and anti-inflammatory activities [9,10,11]. SLPI is widely expressed in tissues and bodily fluids, such as mucus, seminal plasma, cervicovaginal fluid, and amniotic fluid. It modulates inflammation by interfering with IL-8 receptor signaling, contributing to a tissue-protective, anti-inflammatory environment [9]. Recent studies showed that SLPI reduced inflammation and cartilage damage in a bacterial arthritis model [12]. In OA models, SLPI is upregulated in chondrocytes but does not directly modulate progression; it may support regeneration [13].

Although the full spectrum of its extracellular roles remains to be clarified, SLPI holds promise as a therapeutic agent and potential biomarker for OA and other inflammatory disorders [11,13]. Its pleiotropic properties suggest that it may play a pivotal role in promoting tissue repair and resolving inflammation, highlighting its value in the development of novel OA treatments.

## 2. Osteoarthritis Overview

Osteoarthritis (OA) is the most prevalent joint disorder, affecting approximately 10% of men and 18% of women over the age of 60 [1,14]. It is a major public health burden due to its association with chronic pain, disability, reduced quality of life, and increased morbidity and mortality. Particularly in overweight individuals, OA leads to significantly elevated healthcare costs [1]. Despite extensive research efforts, most disease-modifying osteoarthritis drugs (DMOADs) have failed in clinical trials due to insufficient efficacy or unacceptable adverse effects [5,7,8,15]. Consequently, there is growing interest in identifying new therapeutic targets based on a better understanding of OA’s pathophysiology. Promising agents currently in preclinical development target specific molecular pathways contributing to OA’s progression [8,15]. Contrary to its traditional classification as a degenerative joint disease caused solely by mechanical wear, OA is now recognized as a multifactorial condition involving complex interactions between biomechanical forces, inflammatory mediators, genetic predispositions, and systemic metabolic changes [16,17]. Rapid disease onset in some individuals, joint-specific patterns, and systemic features, such as myofascial pain and periarticular tissue remodeling, indicate that both local and systemic factors drive OA’s progression [1,18,19]. A central feature of OA is the breakdown of articular cartilage, which covers the ends of bones in the joint. Knee joint cartilage includes hyaline cartilage (lining femur, tibia, and patella) and fibrocartilage (e.g., the meniscus), both of which are essential for load-bearing and smooth joint articulation [2,10,20,21]. Cartilage is composed mainly of water (65–80%) [22,23], type II collagen in hyaline cartilage, type I collagen in fibrocartilage [20,22,23], and proteoglycans, such as aggrecan, which provides compressive strength through water attraction via glycosaminoglycan (GAG) chains [24,25]. Hyaluronic acid interacts with aggrecan to form large aggregates that further enhance cartilage resilience [25]. Structural glycoproteins like fibronectin and cartilage oligomeric matrix protein (COMP) maintain the integrity of the cartilage matrix [26]. Chondrocytes, the only cellular component of cartilage, synthesize and maintain this matrix [27].

Recent research has highlighted the role of Secretory Leukocyte Protease Inhibitor (SLPI) in OA’s pathogenesis. SLPI, a serine protease inhibitor, is increased in early-stage OA cartilage but diminishes during later stages, potentially leaving chondrocytes vulnerable to proteolytic enzymes [13]. SLPI interferes with LPS/TLR-mediated NF-κB signaling in macrophages, affecting IL-1β responses independently of protease inhibition [28,29,30,31]. SLPI’s regulatory functions in cartilage and inflammation have shown therapeutic potential in animal models of OA, making it a promising target for future drug development. However, SLPI overexpression did not alleviate OA-related pathological phenotypes under conditions like HIF-2α overexpression or DMM (destabilization of the medial meniscus) surgery [13]. Notably, under IL-1β-induced inflammatory conditions, SLPI overexpression reduced the gene expression of MMP3 and MMP13, while SLPI knockdown resulted in increased expression of these matrix-degrading enzymes. These findings suggest that SLPI may not act directly on cartilage-degrading enzymes but rather exert its effects through the regulation of gene expression in target cells.

OA risk is also influenced by a variety of factors, including aging, obesity, genetic background, inflammation, previous joint injuries, and occupation-related stress [1,32,33,34]. Obesity contributes to OA not only through mechanical loading but also by promoting systemic inflammation via adipokines and hormonal imbalances [32,35]. With age, chondrocytes exhibit metabolic shifts and reactivation of fetal-like gene expression programs, altering the extracellular matrix (ECM) and contributing to cartilage degeneration [6,36,37]. Genetic predispositions affecting ECM integrity, including collagen composition and proteoglycan levels, increase susceptibility to OA. Mechanical overload—whether from acute injury or chronic use—can trigger synovial cell proliferation, inflammatory mediator release (e.g., TNF-α, IL-1β), and upregulation of matrix-degrading enzymes, such as MMPs [38,39]. These processes compromise joint stability, damage subchondral bone, and lead to osteophyte formation and cartilage loss [40].

In this complex network of degenerative and inflammatory changes, SLPI plays a unique dual role—protecting cartilage from enzymatic degradation and modulating inflammatory responses. Given its regulation by key cytokines, such as IL-1 and TNF-α [41,42,43,44], both of which are elevated in OA joints, SLPI stands out as a biologically relevant molecule with high therapeutic potential. Enhancing SLPI expression or mimicking its function may represent a novel strategy to counteract OA progression.

## 3. Molecular Basis of SLPI in Osteoarthritis (OA)

Osteoarthritis (OA) is a multifactorial joint disorder characterized by cartilage degradation, subchondral bone remodeling, and chronic inflammation. Genetic predisposition plays a crucial role in OA development, with several genes identified that regulate cartilage integrity, inflammation, and tissue remodeling. Among them are *COL2A1*, encoding type II collagen essential for cartilage structure [36,45]; *MMP13*, which mediates cartilage matrix breakdown [36,37,46]; *GDF5* and *SMAD3*, which influence chondrogenesis and cartilage repair via TGF-β signaling [32,38,39,40]; and *IL1RN*, which modulates inflammatory responses through interleukin-1 antagonism [42].

**Figure 1:**
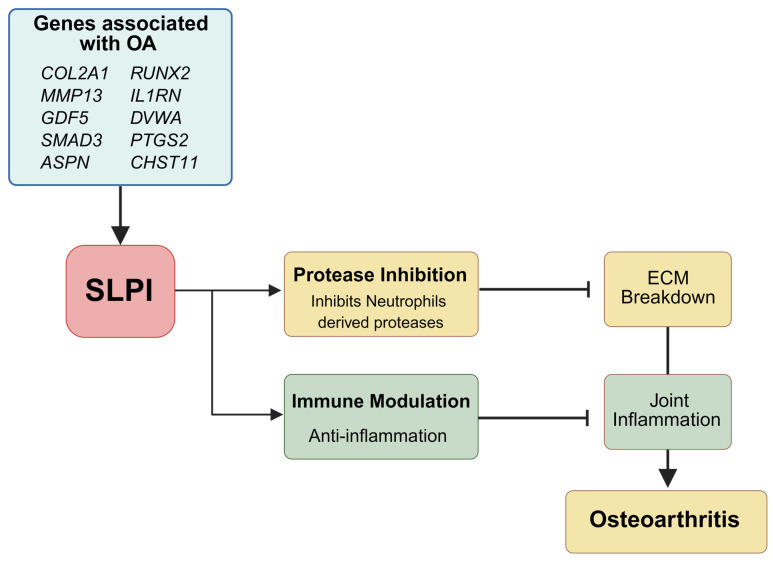
Molecular basis of SLPI in osteoarthritis (OA). This schematic illustrates the interplay between osteoarthritis-associated genes and the biological functions of Secretory Leukocyte Protease Inhibitor (SLPI) relevant to OA’s pathology. Key OA-related genes (*COL2A1*, *MMP13*, *GDF5*, *SMAD3*, *ASPN*, *RUNX2*, *IL1RN*, *DVWA*, *PTGS2*, *CHST11*) regulate cartilage structure, inflammation, and bone metabolism. SLPI, as a serine protease inhibitor, counteracts cartilage matrix degradation by inhibiting neutrophil elastase, cathepsin G, and proteinase 3, and it modulates inflammation through sequestration of inflammatory mediators like serum amyloid A and leukotriene B4. Together, these molecular pathways highlight SLPI’s potential role in preserving joint integrity and regulating inflammation, making it a promising target in OA research. Created in BioRender. Kim, W. (2025) https://BioRender.com/379fyp0.

A gene increasingly recognized in the context of inflammation and tissue homeostasis is *SLPI*. *SLPI* encodes a serine protease inhibitor that regulates innate immune responses and tissue integrity. Its role in OA may be conceptualized through its dual actions: protease inhibition and inflammation modulation (Figure 1).

SLPI inhibits key neutrophil-derived proteases, such as elastase, cathepsin G, and matrix metalloproteinase 3 [47], which, if unregulated, contribute to extracellular matrix (ECM) breakdown, a hallmark of OA pathogenesis. By preserving ECM’s integrity, SLPI acts similarly to tissue inhibitors of metalloproteinases (TIMPs), counteracting catabolic enzymes like MMP13 [48,49,50,51,52]. Furthermore, SLPI’s protease-inhibitory function might be crucial in preventing excessive degradation of cartilage proteoglycans and collagen, paralleling the roles of genes like *CHST11*, which maintains cartilage structure via proteoglycan sulfation [6,43].

Beyond its enzymatic regulation, SLPI exhibits anti-inflammatory properties that are independent of its protease inhibition. It modulates immune cell migration and cytokine signaling by sequestering mediators like serum amyloid A and leukotriene B4, thus dampening neutrophil infiltration and joint inflammation—a process aggravated by IL-1β and TNF-α in OA. SLPI restricts neutrophil migration systemically and at inflammation sites [53]. Serum amyloid A is known to promote pro-inflammatory cytokine release (e.g., IL-1β) and recruit neutrophils via FDRL1 receptors [54,55]. Meanwhile, leukotriene B4 (LTB_4_) is a powerful neutrophil chemoattractant. In OA, IL-1β and TNF-α exacerbate neutrophil-driven inflammatory responses in joints. SLPI’s ability to modulate inflammation complements the genetic contributions of IL1RN and PTGS2 (COX-2) in inflammatory pathways associated with OA [56,57]. SLPI shares functional overlap with IL-1 receptor antagonist (IL-1RN) and COX-2 in terms of controlling inflammation [47]. Genetic polymorphisms in IL1RN and PTGS2 have established roles in OA susceptibility and progression [58,59,60]. A biochemical study found that SLPI is cleaved at two sites in its N-terminal WAP domain, which diminishes its binding to LPS and NF-κB consensus sequences, indicating reduced functional anti-protease/inflammatory modulation [61].

Taken together, the molecular actions of SLPI align closely with key pathogenic mechanisms in OA, including cartilage degradation, inflammation, and tissue remodeling. This makes SLPI a compelling candidate for further research as both a biomarker and a therapeutic target in osteoarthritis.

## 4. SLPI-Mediated Pathways in Osteoarthritis

**Figure 2:**
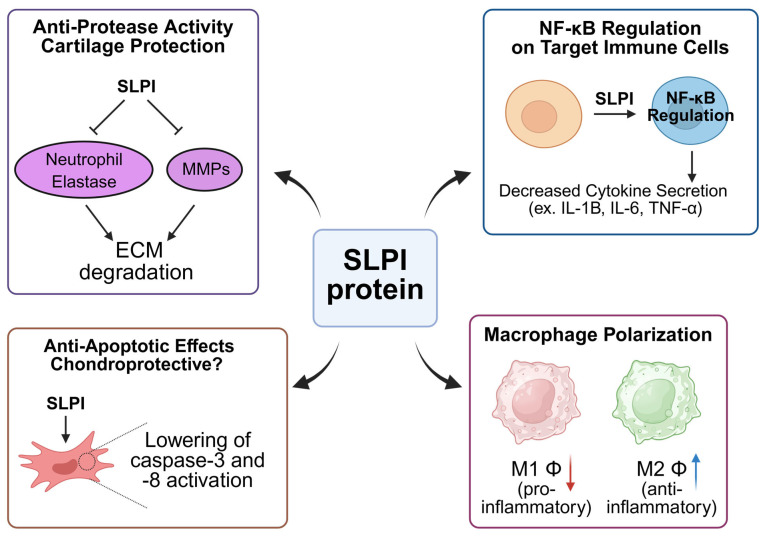
Schematic overview of SLPI-mediated pathways in osteoarthritis. The figure summarizes the protective mechanisms of Secretory Leukocyte Protease Inhibitor (SLPI) in the pathogenesis of osteoarthritis (OA). SLPI inhibits serine proteases, such as neutrophil elastase, cathepsin G, and trypsin, thereby preserving extracellular matrix (ECM) integrity and preventing cartilage degradation. It suppresses the nuclear factor kappa B (NF-κB) signaling pathway, reducing the expression of pro-inflammatory cytokines, including IL-1β, IL-6, and TNF-α, which limits synovial inflammation and matrix metalloproteinase (MMP) activity. SLPI also exerts chondroprotective effects by inhibiting caspase-mediated apoptosis, promoting autophagy, and upregulating anti-apoptotic proteins, such as Bcl-2 and Bcl-xL. Furthermore, SLPI modulates immune responses by downregulating M1 macrophage activation, enhancing M2 macrophage polarization, and regulating T cell responses to maintain joint homeostasis. Collectively, these actions slow OA’s progression and preserve joint structure and function. The red arrows indicate a decrease and blue arrows indicate an increase. Created in BioRender. Kim, W. (2025) https://BioRender.com/6dx8m3w.

Secretory Leukocyte Protease Inhibitor (SLPI) has emerged as a multifaceted modulator in osteoarthritis (OA), acting through anti-inflammatory, anti-protease, chondroprotective, and immunomodulatory pathways (Figure 2). In the degenerative microenvironment of OA—marked by chronic inflammation, cartilage degradation, and synovial hyperplasia—SLPI plays a central role in regulating joint homeostasis.

SLPI is a key serine protease inhibitor that primarily targets neutrophil elastase, cathepsin G, and trypsin—enzymes that degrade cartilage matrix components, such as type II collagen and aggrecan. SLPI’s primary action is inhibiting neutrophil elastase, which degrades tissue, including cartilage ECM, thus safeguarding integrity [28]. SLPI might suppress MMP-3 and MMP-13 expression in chondrocytes under IL-1β, and it might be linked to reduced MMP in arthritis models [12]. SLPI protects proteoglycan and the collagen matrix in joints; intra-articular SLPI protects the cartilage growth factor “Link N” from protease-mediated degradation, which in turn promotes the synthesis of proteoglycans and collagen, maintaining cartilage composition. Moreover, SLPI is one of several anti-angiogenic protease inhibitors expressed by chondrocytes, particularly in the superficial cartilage zone, where it may contribute to the avascular nature of healthy cartilage [62]. Chronic inflammation in OA is driven by pro-inflammatory cytokines like IL-1β, IL-6, and TNF-α, largely regulated through the NF-κB signaling pathway. SLPI inhibits NF-κB signaling, thereby attenuating the transcription of inflammatory genes and MMPs [63,64,65]. A review outlines that IL-1β, TNF-α, and IL-6 drive cartilage inflammation and destruction in OA [66]. A previous study shows that SLPI dampens inflammation tied to these cytokines, reducing protease activity that would damage cartilage [12]. While studies do not explicitly show SLPI reducing cytokine expression, they demonstrate SLPI’s anti-inflammatory effects by restraining their downstream proteolytic activities, effectively suppressing the inflammatory cascade. SLPI mitigates oxidative stress by limiting inflammatory cell activation and reducing reactive oxygen species (ROS) production [67,68]. A broader review highlights SLPI’s cytoprotective functions in non-communicable diseases through tissue regeneration and anti-inflammatory pathways [56]. TNF-α induced cell death and caspase activation [69,70]. SLPI is expressed in synovial fibroblasts and modulates local inflammation and lubrication. SLPI downregulates fibroblast-driven inflammation, reducing the production of cytokines and degradative enzymes [71,72]. SLPI stimulates hyaluronic acid production, enhancing joint lubrication and cushioning [68]. SLPI orchestrates the immune balance between tissue damage and repair. It suppresses M1 pro-inflammatory macrophages while enhancing M2 anti-inflammatory macrophages that aid in tissue repair [73]. SLPI reduces Th17-mediated inflammation and enhances Treg responses, maintaining immune equilibrium in the joint [74,75].

Emerging data suggest that SLPI also plays a role in regulating subchondral bone changes in OA. SLPI supports bone-forming cells, aiding in the maintenance of bone structure beneath damaged cartilage [76,77]. SLPI reduces osteoclast activity, preventing excessive bone resorption associated with OA progression [78]. SLPI acts as a master regulator in OA by simultaneously controlling inflammation, protease activity, apoptosis, immune cell dynamics, and bone remodeling. Its multi-pathway engagement makes it a promising target for future therapeutic interventions aimed at slowing disease progression and preserving joint integrity.

## 5. Preclinical Evidence and Therapeutic Potential of SLPI in Osteoarthritis

The Secretory Leukocyte Protease Inhibitor (SLPI) gene has emerged as a multifunctional regulator implicated in inflammation, tissue remodeling, and immunity, making it an attractive candidate for therapeutic investigation. While SLPI is best known for its anti-inflammatory and antimicrobial roles, increasing evidence suggests that it also participates in bone metabolism and may influence the pathophysiology of degenerative joint diseases, such as osteoarthritis (OA).

### 5.1. SLPI Knockout Models: Functional Insights and Translational Relevance

SLPI knockout (KO) mice have provided valuable mechanistic insights into the gene’s biological function. Although these KO models maintain normal bone homeostasis under basal conditions, they exhibit impaired responses to anabolic stimuli, such as parathyroid hormone (PTH), indicating that SLPI’s activity is context dependent. Notably, SLPI is strongly upregulated in osteoblasts in response to PTH, enhancing osteoblast differentiation and modulating osteoblast–osteoclast interactions to suppress osteoclastic bone resorption [79]. These findings highlight SLPI as a key mediator in bone remodeling under stress or pathological conditions, suggesting its potential as a therapeutic target in diseases characterized by abnormal bone turnover, including OA.

Despite this, current research on SLPI knockout models in OA remains limited. Interestingly, one study found that overexpression of SLPI in experimental OA models did not significantly alter disease progression, implying that SLPI’s role in OA may be more nuanced and possibly reliant on disease stage, local tissue context, or combinatorial interactions with other regulatory molecules, and it revealed that adenoviral SLPI overexpression in OA models did not modify progression, suggesting nuanced, context-dependent effects [13]. These gaps underscore the need for further functional studies, particularly using inducible or tissue-specific knockout models, to determine whether SLPI deficiency contributes to OA’s onset or exacerbation.

### 5.2. Therapeutic Inference and Clinical Potential of SLPI

Parallel to findings from knockout studies, translational research has also pointed toward SLPI as a promising therapeutic node [13,63,80]. SLPI expression varies by tumor type—upregulated and pro-metastatic in some cancers (e.g., breast, gastric, ovarian) while downregulated in others (e.g., certain breast subsets) [81]. Secretory Leukocyte Protease Inhibitor (SLPI) plays a protective role in various diseases by suppressing inflammation, enhancing macrophage-mediated anti-inflammatory responses, improving survival during infections, and contributing to the therapeutic effects of compounds like isorhamnetin and Daikenchuto. Conversely, reduced SLPI expression impairs dental pulp cell activity, suggesting its essential role in maintaining tissue function and immune homeostasis [80,82,83,84]. Histone methylation is regarded as a stable modification important in the epigenetic regulation of gene expression. In addition to epigenetic regulation of constitutive SLPI expression, histone methylation may play a role in stimulated SLPI expression by modulating RNA polymerase recruitment and subsequent gene transcription [85]. Moreover, SLPI has been associated with susceptibility factors in conditions like necrotizing fasciitis and arthritis, reinforcing its role in tissue integrity and immune defense [83,86]. Understanding SLPI’s function, specifically in chondrocytes and synovial fibroblasts, could pave the way for developing targeted therapies aimed at restoring joint homeostasis. Targeting the SLPI signaling axis—either through gene therapy, small molecule activators, or ncRNA modulators—could provide new therapeutic angles for OA, especially in cases where inflammation-driven cartilage degradation predominates [10,11].

Together, SLPI knockout models and transcriptomic profiling converge to underscore the gene’s biological importance and therapeutic promise. While its precise role in OA’s pathology requires further clarification, existing evidence points to SLPI as a context-dependent modulator with druggable potential. Future studies should aim to delineate its downstream signaling cascades, tissue-specific functions, and interactions with epigenetic regulation to fully harness its clinical value in osteoarthritis and beyond.

## 6. SLPI Expression in Human Osteoarthritis (OA) Samples

**Figure 3:**
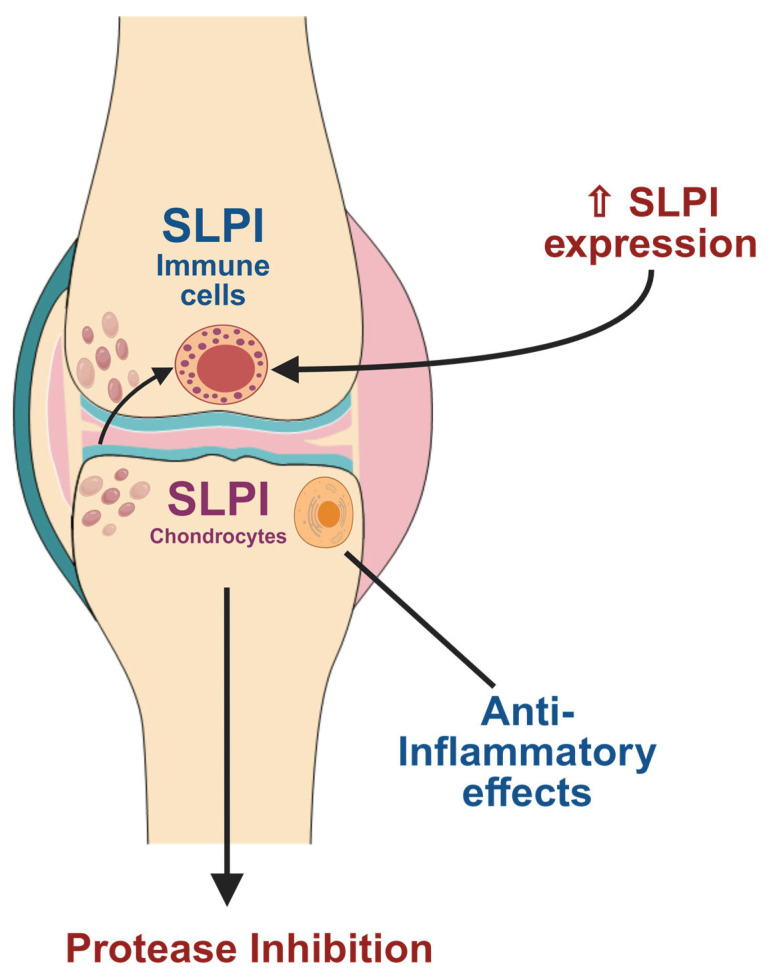
SLPI expression in human OA joints. Illustration of an osteoarthritic knee joint showing the synovial lining, synovial fluid, and articular cartilage. Increased SLPI expressions are indicated in synovial fibroblasts, chondrocytes, and infiltrating immune cells. Arrows highlight their protective functions, including protease inhibition and suppression of inflammation. Created in BioRender. Kim, W. (2025) https://BioRender.com/u9pzhkp.

Secretory Leukocyte Protease Inhibitor (SLPI) has been increasingly implicated in the pathophysiology of OA. Recent transcriptomic and proteomic analyses of human synovial tissues and cartilage from OA patients reveal that SLPI expression is significantly upregulated in affected joints compared to non-OA controls (Figure 3). Transcriptomic and proteomic analyses show that SLPI is upregulated in OA-affected cartilage or joints compared to controls. A review of multi-omics in OA states that SLPI is among the most highly secreted proteins in OA cartilage [87]. For instance, a previous study showed elevated SLPI mRNA and protein levels in synovial tissues from OA patients, suggesting an endogenous protective response against tissue degradation and inflammation [13]. Furthermore, proteomic profiling of synovial fluid has identified SLPI as one of the differentially expressed proteins correlating with OA severity, highlighting its translational potential as both a biomarker and therapeutic target.

## 7. Comparative Insights: SLPI’s Role in Other Inflammatory Conditions

Beyond OA, SLPI plays a crucial role in modulating inflammation across various tissues. In chronic obstructive pulmonary disease (COPD), SLPI is highly expressed in the airway epithelium, where it neutralizes neutrophil elastase, reducing tissue damage (Table 1) [88]. Similarly, in inflammatory bowel disease (IBD), SLPI expression is induced in the intestinal mucosa, where it mitigates protease-mediated injury and promotes mucosal healing [89,90]. These observations suggest that SLPI acts as a conserved anti-inflammatory and tissue-protective factor across diverse chronic inflammatory settings. Thus, understanding its regulation and function in OA may offer insights into shared mechanisms of inflammatory tissue remodeling and open avenues for repositioning SLPI-modulating therapies.

## 8. Emerging Therapies or Biotech Developments Involving SLPI

Recent biotechnological efforts have aimed to harness SLPI’s anti-protease and anti-inflammatory functions for therapeutic applications. Recombinant SLPI (rSLPI) has been explored in preclinical models of arthritis, where intra-articular administration attenuates cartilage erosion and reduces inflammatory cytokine production [12]. Biotech companies are also developing SLPI-derived peptides and mimetics to selectively inhibit pathogenic proteases while minimizing off-target effects. Such developments not only strengthen the therapeutic rationale for SLPI in OA but also suggest potential combinatorial strategies with current disease-modifying OA drugs.

## 9. Discussion

Secretory Leukocyte Protease Inhibitor (SLPI) has emerged as a key endogenous regulator of inflammation, protease activity, and immune responses—hallmarks of osteoarthritis (OA) pathophysiology. Its multifaceted role in cartilage protection, chondrocyte survival, immune modulation, and joint homeostasis underscores its therapeutic promise in OA. Despite considerable progress, several critical gaps and controversies remain, necessitating deeper mechanistic and translational exploration.

One of the most prominent pathological features of OA is the degradation of the cartilage extracellular matrix (ECM), driven largely by elevated protease activity, including matrix metalloproteinases (MMPs) and aggrecanases [46]. A detailed biochemical characterization confirms that SLPI is a potent reversible inhibitor of neutrophil elastase (HNE), cathepsin G (CatG), chymase, chymotrypsin, and trypsin—achieving inhibition constants in the nanomolar to picomolar range [29,50,97,98]. SLPI significantly suppressed the production of MMPs, including MMP-2, MMP-3, and MMP-9, in monocytes. In chondrocyte-focused arthritic models, SLPI also reduced MMP-3 and MMP-13 levels, dampening the enzymatic breakdown of the cartilage extracellular matrix [99]. These anti-protease effects position SLPI as a critical defender of ECM integrity and a potential candidate for halting OA progression.

In addition to its anti-proteolytic function, SLPI exerts potent anti-inflammatory effects by modulating the NF-κB pathway—a central mediator of inflammation [63,100].

Furthermore, SLPI contributes to chondroprotection by preventing chondrocyte apoptosis and promoting autophagy. Apoptotic cell death in chondrocytes is a key contributor to irreversible cartilage loss. These findings suggest that SLPI could preserve chondrocyte viability, thus maintaining the structural and functional integrity of cartilage.

Beyond cartilage, SLPI appears to participate in subchondral bone remodeling. Recent studies have shown that SLPI is upregulated by parathyroid hormone (PTH), and its presence is essential for PTH-induced osteoblast differentiation and bone formation [79]. This expands SLPI’s potential therapeutic relevance from cartilage protection to overall joint preservation, particularly in early-stage OA, when subchondral changes are prominent.

These immune-balancing effects could be instrumental in limiting chronic inflammation and tissue damage in OA joints.

Despite its promising biological activities, several key research gaps and challenges persist. The exact molecular interactions between SLPI and components of the NF-κB pathway and MMPs remain incompletely defined. Moreover, while compelling evidence exists from in vitro and animal models, there is a paucity of human clinical data regarding SLPI expression levels in OA patients and its correlation with disease severity.

Even with the evidence that revealed that SLPI exhibits anti-inflammatory and tissue-protective properties in vitro and in various disease models, a notable study demonstrated that chronic overexpression of SLPI did not protect mice from developing osteoarthritis (OA) in the destabilization of the medial meniscus (DMM) model [13]. This finding raises important questions regarding the timing, localization, and context-dependent functionality of SLPI in OA’s pathogenesis. Several potential explanations may account for this apparent contradiction. SLPI’s function may be context specific, requiring tightly regulated expression in response to acute injury or inflammatory signals rather than constitutive overexpression. In normal physiological conditions, excessive SLPI may disrupt the homeostatic balance of protease activity, potentially hindering tissue remodeling or leading to compensatory mechanisms that reduce its efficacy [101]. It is possible that SLPI’s protective effects are more effective during specific windows of OA progression, such as early inflammation, rather than during chronic, structural degradation. Overexpression throughout the organism may not mimic the joint-specific, cell-type-specific expression patterns needed for therapeutic benefit. For instance, SLPI expression is often seen in synovial fibroblasts and chondrocytes, where it regulates matrix metalloproteinase activity and inflammatory responses [73].

Our findings and synthesis highlight SLPI’s upregulation in human OA joints, consistent with its protective roles observed in other chronic inflammatory diseases, such as COPD and IBD. This cross-disease comparison underscores the conserved nature of SLPI’s anti-protease and immunomodulatory activities, suggesting potential shared therapeutic strategies. Importantly, recent developments in SLPI-based therapeutics—including recombinant proteins, viral gene delivery, and small molecule mimetics—offer promising avenues for translational application in OA. Incorporating SLPI modulation into current OA treatment regimens could address the unmet need for disease-modifying interventions, paving the way for precision medicine approaches targeting inflammatory joint disease.

Another major frontier is therapeutic delivery. While recombinant SLPI and gene therapy approaches show promise, issues surrounding delivery efficiency, tissue targeting, and long-term expression remain unresolved. Nanoparticle-mediated delivery systems and CRISPR-based SLPI modulation are exciting avenues that warrant rigorous investigation.

Finally, the potential of SLPI as a biomarker for OA diagnosis and progression is underexplored. Correlating SLPI levels in synovial fluid or serum with OA stages could pave the way for its use as a diagnostic or prognostic marker, offering a more personalized approach to disease management.

## 10. Conclusions

Osteoarthritis remains a significant clinical challenge due to its multifactorial etiology and lack of effective disease-modifying treatments. The Secretory Leukocyte Protease Inhibitor (SLPI) stands out as a compelling therapeutic candidate and biomarker, given its unique combination of anti-proteolytic, anti-inflammatory, chondroprotective, and immunomodulatory properties.

By preserving the cartilage matrix, suppressing inflammatory cascades, preventing chondrocyte death, and modulating immune responses, SLPI orchestrates a comprehensive defense against the progression of OA. Moreover, its involvement in bone remodeling and immune regulation broadens its relevance beyond cartilage, suggesting that SLPI may be central to maintaining overall joint health.

Translating these insights into clinical applications will require an integrated effort involving mechanistic studies, innovative therapeutic delivery platforms, and well-designed human clinical trials. With advancements in molecular biology, gene editing, and nanotechnology, SLPI-based therapies hold promise to revolutionize OA treatment by shifting the paradigm from symptom relief to true disease modification.

Future research should continue to dissect SLPI’s molecular networks, explore its interaction with the joint microenvironment, and develop precision-targeted therapeutics that leverage its multifactorial benefits. Harnessing the full therapeutic potential of SLPI could mark a turning point in OA management, offering patients not only symptom relief but also long-term joint preservation.

## Figures and Tables

**Table 1:** Summary of SLPI expression and function in inflammatory diseases.

Disease	SLPI Expression Pattern	Protective Functions	Reference
Osteoarthritis (OA)	Upregulated in chondrocytes and synovial tissue but may not modulate disease progression	Potential biomarker; inhibits neutrophil elastase activity in cartilage	[13]
Rheumatoid Arthritis (RA)	Elevated in synovium and macrophages; induced by TNF-α and IL-1β	Protects against protease-mediated damage; inhibits NF-κB activation	[91]
Chronic Obstructive Pulmonary Disease (COPD)	Upregulated in airway epithelial cells and alveolar macrophages	Inhibits neutrophil elastase; mitigates airway inflammation	[92]
Cystic Fibrosis (CF)	Highly expressed but often inactivated by neutrophil elastase	Antimicrobial, anti-elastase; protects airway lining	[93]
Sepsis	Elevated in plasma during severe infection	Limits systemic inflammation; correlates with organ function	[93]
Inflammatory Bowel Disease (IBD)	Upregulated in colonic epithelium in active disease	Protects mucosa; suppresses cytokines; aids barrier repair	[94]
Psoriasis	Increased in keratinocytes; affects neutrophil responses	Reduces skin inflammation, dryness; regulates NETs	[95]
Periodontitis	High in gingivitis, decreased in chronic periodontitis	Inhibits local proteases and bacterial damage	[96]

## Data Availability

No new data were created or analyzed in this study.
